# Case Report: Pediatric Patient with COVID-19 and Multisystem Inflammatory Syndrome in Children

**DOI:** 10.5811/cpcem.2020.8.48376

**Published:** 2020-09-08

**Authors:** MacKenzie W. Burger, Marcus A. Moore, John M. Wilburn

**Affiliations:** Wayne State University School of Medicine, Detroit Medical Center Sinai-Grace Hospital, Department of Emergency Medicine, Detroit, Michigan

**Keywords:** COVID-19, Kawasaki Disease, PMI, ECMO

## Abstract

**Introduction:**

Coronavirus disease 2019 (COVID-19) rarely manifests with severe complications in pediatric patients. An association between COVID-19 and a Kawasaki-like inflammatory syndrome has recently presented in pediatric patients.

**Case Report:**

We report a unique case of multisystem inflammatory syndrome in children presenting with characteristic findings in a child who later developed cardiogenic shock requiring venoarterial extracorporeal membrane oxygenation.

**Conclusion:**

Recognition of these early signs and symptoms facilitates screening and risk stratification of pediatric COVID-19 cases associated with increased morbidity.

## INTRODUCTION

The coronavirus disease 2019 (COVID-19) pandemic has caused more than 21.7 million confirmed cases and 771,000 deaths worldwide as of August 17, 2020.^1^ Adult patients comprise the majority of serious cases with acute respiratory failure contributing to significant morbidity and mortality. Pediatric cases of COVID-19 are often associated with mild illness or an absence of symptoms, with critical pediatric cases causing respiratory failure or multiple organ dysfunction syndrome in less than 1% of patients.^2^ We describe a case of a pediatric patient with COVID-19 who initially presented to the emergency department (ED) with signs of an early developing atypical Kawasaki disease (KD) vs streptococcal pharyngitis and who later developed multisystem inflammatory syndrome in children (MIS-C) with myocarditis and cardiogenic shock requiring venoarterial extracorporeal membrane oxygenation (VA-ECMO).

Recent literature has identified cases of a syndrome similar to KD occurring concurrently with or subsequent to COVID-19 in a vulnerable pediatric cohort, with an association between severe acute respiratory syndrome coronavirus 2 (Sars-CoV-2) and inflammatory vasculitis.^3^ As of August 2020, there were 570 confirmed MIS-C cases in 40 states, with 10 reported deaths.^4^ ECMO was used in a smaller percent of patients, with a case series of 186 MIS-C cases reporting VA-ECMO use in eight patients.^5^ The majority of MIS-C cases are reported in medicine and pediatric journals from the perspective of authors who did not interact with the patient early in his or her disease course in the ED, with sentinel signs days before decompensation. This is a case report of a pediatric patient with COVID-19 developing myocarditis and cardiogenic shock with cardiac arrest and subsequent VA-ECMO use following an initial presentation of suspected early KD vs early toxic shock syndrome now increasingly recognized as a separate inflammatory syndrome in pediatric COVID-19 cases. Recognition of this pro-inflammatory phenotype, MIS-C, is necessary to effectively risk stratify pediatric patients with COVID-19.

## CASE REPORT

During the COVID-19 pandemic, a six-year-old Black female presented to an urban ED after an episode of syncope with two days of subjective fevers, sore throat, abdominal pain, and a newly developing rash. History was obtained from her parents, who reported loss of appetite and increased fatigue. Her parents were treating her fever with acetaminophen, but they became concerned when the patient had a brief episode of syncope when attempting to stand. Prior to this illness, she had no remarkable past medical history and was up to date with vaccinations. Family was unaware of any sick contacts.

The patient was tired on examination, but appropriately interactive. She had a fever of 39.5° Celsius (C) and was tachycardic with a heart rate of 138 beats per minute (bpm). Other vitals were unremarkable. Her fever defervesced to 36.8°C with acetaminophen and ibuprofen, and the heart rate subsequently normalized. An electrocardiogram (ECG) was not obtained due to clinical impression that orthostasis was secondary to dehydration. The abdominal examination was benign, her throat was minimally erythematous, and her lips were cracked. She did not have lymphadenopathy. Streptococcal polymerase chain reaction (PCR) testing (rapid strep) was positive. On reevaluation, a blanchable erythematous rash initially present on her hands and abdomen had spread to her bilateral lower extremities and the dorsum of her feet. A diagnosis of early KD was considered due to the high fever and cutaneous symptoms, but the patient did not meet criteria for diagnosis. She was given amoxicillin and close follow-up.

The patient returned to the ED three days later with persistent fever and periumbilical abdominal pain, as well as new-onset difficulty breathing, bilateral conjunctivitis, and swollen hands. She had delayed capillary refill, and she was tachypneic and hypotensive with a blood pressure of 70/40 millimeters of mercury (mm Hg). An ECG revealed a heart rate of 116 bpm (reference [ref] range 75–115 bpm) with a prolonged PR interval of 188 milliseconds (ms) (90–170 ms) consistent with first-degree atrioventricular block (Image). Labs were pertinent for ferritin 699 nanograms (ng) per milliliter (mL) (ref: 11–306.8 ng/mL); albumin 3.8 gram (gm) per deciliter (dL) (ref: 3.8–4.7 gm/dL); lactate dehydrogenase 794 units (U) per liter (L) (ref: 140–271 U/L); blood urea nitrogen 33 milligrams (mg)/dL (ref: 7–25 mg/dL); creatinine 1.09 mg/dL (ref: 0.3–0.6 mg/dL), high-sensitivity troponin 114 ng/L (ref: 3–17 ng/L), D dimer 4.21 mg/L (ref: <0.5 mg/L); fibrinogen 834 mg/dL (ref: 186–466 mg/dL); C-reactive protein 450 mg/L (ref: <5.0 mg/L); and hyponatremia with a serum sodium of 118 millimoles (mmol)/L (ref: 136–145 mmol/L). Central and arterial lines were placed, and epinephrine and dopamine drips were started after point-of-care ultrasound revealed diminished left heart function.

A comprehensive cardiac echocardiogram (echo) was obtained upon admission that revealed mildly decreased left ventricular (LV) function, septal hypokinesis, and mild mitral valve (MV) insufficiency with an ejection fraction (EF) of 59%. There were no coronary artery aneurysms. She was admitted to the pediatric intensive care unit for cardiogenic shock vs septic shock from KD vs toxic shock syndrome. High-dose aspirin 10 mg/kilogram (kg) every six hours by mouth and intravenous (IV) immunoglobulin 2 cubic centimeter (cc)/kg were initiated along with broad-spectrum antibiotics. COVID-19 PCR testing was positive. Overnight, the patient became increasingly hypoxic and had an accelerated junctional rhythm before developing a bradyarrhythmia, seizure-like activity, and pulseless electrical activity cardiac arrest with return of spontaneous circulation in five minutes. Patient was intubated, sedated, and had several runs of non-sustained ventricular tachycardia before VA-ECMO therapy was initiated for cardiogenic shock.

CPC-EM Capsule

What do we already know about this clinical entity?

*Multisystem inflammatory syndrome in children (MIS-C) case definition: a patient <21 years with fever, inflammation, severe illness requiring hospitalization with multisystem involvement and current/recent coronavirus disease 2019*.

What makes this presentation of disease reportable?

*Spanning two emergency department visits with a worsening clinical presentation, this case of MIS-C is an example of what not to miss*.

What is the major learning point?

*Awareness of early symptoms and screening labs are key as most MIS-C patients recover with appropriate intervention*.

How might this improve emergency medicine practice?

*Familiarizing physicians with MIS-C and appropriate screening labs will heighten awareness when potential MIS-C patients present*.

Patient was successfully taken off ECMO after a course of approximately six days. LV dysfunction and renal failure resolved with repeat comprehensive echo revealing mild concentric LV hypertrophy, normal LV function, trivial MV insufficiency, and mild pulmonary valve insufficiency with an EF of 69%. On discharge, neurological function was within normal limits and consistent with baseline mentation.

## DISCUSSION

Pediatric patients presenting with febrile illness are common, similar to this patient who was initially treated symptomatically and given appropriate medical follow-up prior to decompensating days later. However, during the COVID-19 pandemic, it is imperative that emergency physicians effectively screen non-toxic appearing, febrile pediatric patients for indicators of MIS-C. Our patient was not tested for COVID-19 at the time of the initial encounter for several reasons. COVID-19 PCR testing was restricted to patients with hypoxia and clinical presentations consistent with the known disease at the time, which our patient lacked. Furthermore, testing of pediatric patients was not routine due to lack of known pediatric morbidity and mortality. We now recognize that febrile pediatric patients should undergo COVID-19 testing, but MIS-C can still present with a negative PCR screen and positive immunoglobulin G antibodies following an acute infection. A negative COVID-19 screen also does not exclude the diagnosis due to the imperfect sensitivity of the PCR testing.^5^

What is now being referred to as MIS-C was unknown at time of treatment. We considered developing KD as an etiology; however, the patient did not have five days of fever or four of the five required additional criteria for diagnosis. On the initial visit, our febrile patient had a positive streptococcal PCR test and complaints of abdominal pain and a sore throat, so she was treated for streptococcal pharyngitis. However, clinically the diagnosis of streptococcal pharyngitis was unconvincing, as she had an unremarkable pharyngeal examination. Similar cases of COVID-19-positive pediatric patients were being reported weeks later around the United States. In retrospect, our patient was fitting a similar pattern presenting rarely in pediatric patients with COVID-19.^3^

KD, or mucocutaneous lymph node syndrome, is an inflammatory disease of the middle-sized blood vessels with a severe complication of coronary artery aneurysms. There is no single test that identifies KD; rather it is a clinical diagnosis that most commonly affects children younger than five years of age. Classic KD requires fever of five days with four of the five following criteria: bilateral conjunctivitis; a maculopapular rash; mucous membrane changes; cervical adenopathy; and edema or erythema of the hands and feet.^6^ Incomplete KD is defined as fever of five days with fewer than four diagnostic criteria. Many experts also believe that KD can be diagnosed in the presence of classic features with fewer than five days of fever by experienced clinicians. Both complete and incomplete KD are complicated by coronary aneurysms, so it is important for incomplete KD to remain on the differential. Another variation, KD shock syndrome (KDSS), refers to KD patients with greater than 20% decrease in systolic blood pressure.^7^ On repeat presentation, our patient met criteria for classic KD and KDSS and received treatment with IV immunoglobulin (IG) and high-dose aspirin therapy.

Toxic shock syndrome and KDSS can be difficult to distinguish, as both present with shock, fever, and a rash. Our patient was treated with broad-spectrum antibiotics including clindamycin for streptococcal exotoxin along with receiving IVIG and high-dose aspirin; this was continued until initiation of VA-ECMO, which requires heparinization. Echocardiography can help with differentiation, as tricuspid regurgitation, mitral regurgitation, and coronary artery dilation are associated with KD.^7^ The average age of patients also varies by diagnosis. KD most commonly affects children younger than five years old. The typical age for pediatric toxic shock syndrome is younger than two years, and MIS-C affects patients younger than 21, with an average age of eight.^5^

Viral infections have been hypothesized to incite the cytokine storm and inflammatory changes that characterize KD. Inflammatory markers are also used as a prognostic factor in COVID-19. Elevated d-dimer (>3,000 ng/mL fibrinogen equivalent units), C-reactive protein (>3 mg/dL), B-type natriuretic peptide (>400 picograms/mL), and increased fibrinogen (>400 mg/dL) are associated with more severe presentations, and most MIS-C cases have elevations in at least four of these inflammatory biomarkers.^5^,^8^ Elevations in troponin, creatine phosphokinase, lactate dehydrogenase, low albumin, and hyponatremia have also been present in other patients with COVID-19 positive MIS-C. Obtaining laboratory studies of these markers and echocardiography may help identify and risk stratify pediatric patients with COVID-19 and early presentations of MIS-C.

Non-toxic appearing pediatric patients with a fever for more than 48 hours and a rash or gastrointestinal symptoms should be screened for MIS-C. Appropriate screening labs are complete blood count, C-reactive protein, comprehensive metabolic panel, d-dimer, ferritin, fibrinogen, prothrombin time, partial thromboplastin time, international normalized ratio B-type natriuretic peptide, and troponin. Laboratory tests to evaluate other etiologies should also be obtained and include urinalysis, urine cultures, blood cultures, and viral studies as warranted. Patients should be admitted for further evaluation and management if any of these laboratory findings are abnormal. Laboratory studies indicative of severe disease include hyponatremia, elevations in d-dimer and C-reactive protein levels, increased ferritin, and evidence of myocardial injury or multiple organ dysfunction syndrome. First-degree heart blocks, which this patient presented with, may also be seen in pediatric patients with myocarditis.^9^ Sinus tachycardia, arrhythmias, and non-specific changes are electrocardiography findings reported with MIS-C.

## CONCLUSION

In the era of COVID-19, it appears that there are multiple locations where multisystem inflammatory syndrome in children is affecting pediatric patients who are COVID-19 positive.^3^,^10^ We recommend laboratory screening, cardiac monitoring, acetaminophen for fever, an ECG, and cardiac point-of-care ultrasound in the ED for patients in which this syndrome is suspected. Toxic-appearing pediatric patients with suspected MIS-C should also receive early vasopressors, IVIG, judicious fluids, and broad-spectrum antibiotics in addition to early intensive care unit and cardiology consultations and transfer to a facility with extracorporeal membrane oxygenation capability. Early identification, prognostication, and treatment of COVID-19 positive patients with MIS-C requires further research.

## Figures and Tables

**Image:**
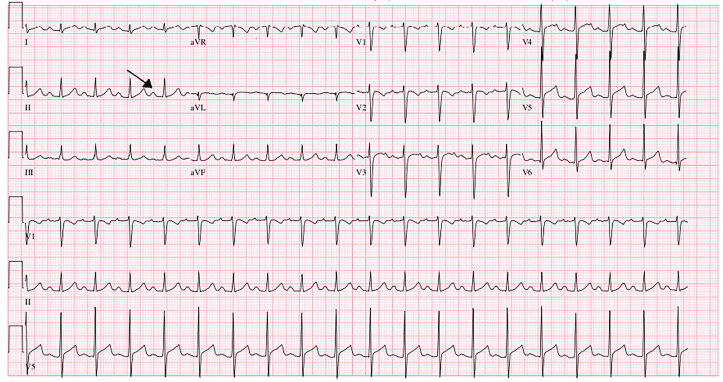
Electrocardiogram of a six-year-old female with multisystem inflammatory syndrome in children reveals first-degree atrioventricular block identified by arrow in lead II.
